# Supplementary material for: Gender and racial bias issues in a commercial “tone of voice” analysis system
Source: PLoS One. 2025 Feb 28;20(2):e0314470. doi: 10.1371/journal.pone.0314470 (PMC11870350; doi:10.1371/journal.pone.0314470)
Supplement: Attachment — Submitted filename: Response Letter-Halo Revisions.docx [file pone.0314470.s001.docx]

Revisions PONE-D-24-23257

Reviewer 1

| Comment | How was it addressed? |
| --- | --- |
| But I think the article would be a better contribution if the authors said more about why the bias that they demonstrate is a significant detriment to well-being. "Employment evaluation" is tossed off in the last sentence as an afterthought. Was Halo used in this way? The only real explanation of the connection to health or well-being is the one the authors cite from the manufacturer, but there is no analysis as to whether this explanation corresponds at all to the reality of the use of Halo. Why, practically, would a Black woman, for example, be particularly concerned about the bias demonstrated here? | We have elaborated the points related to potential harms across domains, including a discussion of the use of similar technologies in healthcare and employment. We have explained that though the Halo is one consumer-facing device, similar technologies are being deployed at a large scale in everything from mental health evaluation to AI-based speech therapy. A Black woman, for example, should be concerned that such technologies are more likely to negatively evaluate them as compared to a similar white speaker, thus misdiagnosing their illness or lowering their job performance ratings. |
| Authors have not made all data underlying the findings in their manuscript fully available? | Our IRB approval does not allow us to make data publicly available without institutional co-agreements. Data is available upon request to researchers who contact us and can agree to the provisions laid out by the IRBs applications for this project from both Pomona College and the University of Alabama. |
| In addition, the authors focus on a product that no longer exists but then conclude in a sweeping way that their findings are pertinent for a range of products still in use. That statement should be explained. Are all the algorithms associated with these products so similar? | See above. Additionally, we have also more clearly articulated the LLM technology that underlies the Halo, and how similar systems are used by companies such as Zoom (for employment evaluation) and Ambiq (for mental health diagnosis).  Indeed, all of these systems rely on ASRs that run LLMs, using machine learn algorithms that have scraped massive, decontextualized data sets. The racial bias in LLMs has been shown across nearly every system that has been tested, and they are based upon the training data that each of these companies use. We have added some references to and discussion of Hoffman et al (2024) that further explains these points. |
| Minor points: --The term "L2 English speaker" should be defined. --There is inconsistent capitalization of "white" and capitalizations of terms such as "race" and "gender" that do not warrant capitalization. --There is incorrect hyphenization of "the speaker side of the..." and of "speech signal" when they are used as nouns and not compound adjectives, but the hyphen is missing in the compound adjective "context-dependent", for example. | Minor points have been addressed. |
|  |  |

Reviewer 2

| Comment | How was it addressed? |
| --- | --- |
| It isn't clear what questions were being asked (or hypotheses tested - either frame would help) | We made explicit the research questions that we were exploring in this paper - that of determining if systematic bias existed in the Halo scores based on race and/or gender. Further, we made clear our hypothesis, based on background information from our literature review. |
| Clarify the purpose of the acoustic analysis, provide a table with the names of the measures completed (in addition to abbreviations), the purpose of segmenting the utterances (and why it matters that you could not determine how HALO divided them). | We created a table that explained the abbreviations and grouped the measures according to what types of speech and vocal phenomena they are used to measure. We also included discussion of why the sentences needed to be segmented, and the reason for why our inability to determine how Halo segmented them was important. |
| Explain the regression + t-test decisions. What were you testing with each? | We included statements about why each t-test was performed. |
| The presentation of the results did not seem to address all of the questions it appeared you were asking. | With the addition of clear research questions and hypothesis, the results are now more clearly motivated - were there systematic biases in the Halo scores based on race and/or gender. Our findings are that there were, and thus our results are in line with our questions. |
| Because the results of the regressions were not quite clear and the values so close, the discussion seemed more definite than the data would lead one to see. | We have made an effort to be more nuanced in our presentation of the results, given the regression outputs. |
| Side note: Figure 1 was not legible on either my monitor or when printed. | Figure 1 is legible on our computers. |
